# Induced pluripotent stem cell-derived mesenchymal stem cells deliver exogenous miR-105-5p via small extracellular vesicles to rejuvenate senescent nucleus pulposus cells and attenuate intervertebral disc degeneration

**DOI:** 10.1186/s13287-021-02362-1

**Published:** 2021-05-13

**Authors:** Yongjin Sun, Wenzhi Zhang, Xu Li

**Affiliations:** Spine Center, Department of Orthopaedics, The First Affiliated Hospital of USTC, Division of Life Sciences and Medicine, University of Science and Technology of China, No.17, Lujiang Road, Hefei, Anhui 230001 People’s Republic of China

**Keywords:** Stem cell, Small extracellular vesicles, miR-105-5p, Nucleus pulposus cells, Intervertebral disc degeneration

## Abstract

**Background:**

Mesenchymal stem cell-derived small extracellular vesicles (MSC-sEVs) have emerged as a promising new therapeutic strategy for intervertebral disc degeneration (IVDD). However, the drawbacks of MSCs, including their invasive access, the donor age, and their limited proliferative capacity, hinder the quantity and quality of MSC-sEVs. Induced pluripotent stem cell-derived MSCs (iMSCs) provide an indefinite source of MSCs with well-defined phenotype and function. This study aimed to investigate the therapeutic effect of sEVs derived from iMSC (iMSC-sEVs) on IVDD and explore the underlying molecular mechanisms.

**Methods:**

IVDD models were established by puncturing discs from the tails of rats. Then, iMSC-sEVs were injected into the punctured discs. The degeneration of punctured discs was assessed using MRI and HE and immunofluorescence staining. The age-related phenotypes were used to determine the effects of iMSC-sEVs on senescent nucleus pulposus cells (NPCs) in vitro. Western blotting was used to detect the expression of Sirt6. miRNA sequencing analysis was used to find miRNAs that potentially mediate the activation of Sirt6.

**Results:**

After intradiscally injecting iMSC-sEVs, NPC senescence and IVDD were significantly improved. iMSC-sEVs could rejuvenate senescent NPCs and restore the age-related function by activating the Sirt6 pathway in vitro. Further, microRNA sequence analysis showed that iMSC-sEVs were highly enriched in miR-105-5p, which played a pivotal role in the iMSC-sEV-mediated therapeutic effect by downregulating the level of the cAMP-specific hydrolase PDE4D and could lead to Sirt6 activation.

**Conclusion:**

iMSC-sEVs could rejuvenate the senescence of NPCs and attenuate the development of IVDD. iMSC-sEVs exerted their anti-ageing effects by delivering miR-105-5p to senescent NPCs and activating the Sirt6 pathway. Our findings indicate that iMSCs are a promising MSC candidate for obtaining sEVs on a large scale, while avoiding several defects related to the present applications of MSCs, and that iMSC-sEVs could be a novel cell-free therapeutic tool for the treatment of IVDD.

## Introduction

Intervertebral disc degeneration (IVDD) is a widely recognized contributor to lower-back pain. IVDD increases both the burden imposed on global health care systems and the risk of disability [1]. An IVD is comprised of an inner nucleus pulposus (NP) surrounded by the annulus fibrosus. NP cells (NPCs) located in the inner NP are responsible for producing a gelatinous extracellular matrix that is composed of collagen II and proteoglycan, among others, which enable the disc to cope with diverse external mechanical stimuli [2]. Accumulating evidence indicates that the senescence of NPCs plays a key role in the pathological progression of IVDD [3]. These senescent NPCs show distinct catabolic features characterized by a decreased proliferation capacity, the loss of functional capability, and an increased secretion of a senescence-associated secretory phenotype [4]. In addition, senescent NPCs also reinforce senescence in an autocrine manner or affect tissue homeostasis in a paracrine manner, leading to a vicious cycle of local catabolism and inflammation. Therapeutic strategies designed to ameliorate the senescence of NPCs should effectively delay the progression of IVDD.

Recently, mesenchymal stem cell (MSC) transplantation has shown promising therapeutic potential in alleviating ageing-associated phenotypes [5, 6]. Despite their potential therapeutic applications, the direct use of stem cell transplantation still faces several hurdles, such as the risk of tumorigenesis and undesirable immune responses [7]. Recent evidence has indicated the therapeutic potential of small extracellular vesicles (sEVs) secreted by MSCs derived from different tissues in alleviating cellular senescence, while avoiding the undesirable immune response and the risk of tumorigenesis [8, 9]. However, harvesting MSCs from different tissues, such as the bone marrow and adipose tissue, is invasive. In addition, limitations such as the decreased proliferative potential and therapeutic efficacy of MSCs during in vitro expansion have impeded the industrial production of sEVs [10].

Induced pluripotent stem cells (iPSCs) are a subpopulation of stem cells that can be reprogrammed from any tissue type in the body. iPSCs have a unique ability to proliferate indefinitely and display totipotency in vitro [11, 12]. MSCs derived from iPSCs (iMSCs) could be expanded in vitro over 40 passages with high efficiency [13]. Furthermore, iMSCs possess MSC-like therapeutic effects in tissue regeneration treatments [14, 15]. Along with the advantages of the acquisition and proliferation of iMSCs, compared with those of MSCs, sEVs can be abundantly obtained from iMSCs, which is convenient for industrial production.

The therapeutic effects of iMSC-derived sEVs (iMSC-sEVs) on cellular senescence are unclear. We speculated that, similar to MSC-sEVs, iMSC-sEVs might also have anti-senescence functions. Since iMSC-sEVs can be harvested infinitely from iMSCs, the demonstration of an anti-senescence function was explored in this study, with the goal of developing an improved IVDD treatment.

## Materials and methods

### Cell culture

A human iPSC line, which was purchased from the Cell Bank of the Chinese Academy of Sciences, was cultured in mTeSR1 medium (STEMCELL Technologies). The iMSCs were transduced as previously described [16]. Briefly, a mTeSR1 medium was replaced with Dulbecco’s modified Eagle medium (DMEM, Hyclone) supplemented with 10% foetal bovine serum (FBS; Sigma-Aldrich), 1% penicillin/streptomycin, 2 mM L-glutamine, and 0.1 mM non-essential amino acids (Gibco). These cells were continuously passaged in the MSC medium until they developed a homogeneous fibroblastic morphology. Human dermal fibroblasts (FBs) were purchased from the Cell Bank of the Chinese Academy of Sciences and were cultured in high-glucose DMEM (Hyclone) with 10% FBS (Sigma-Aldrich) and 1% penicillin/streptomycin. Human NPCs were isolated from normal NP tissue derived from lumbar trauma patients who underwent spinal fusion with no degenerative signal observed upon magnetic resonance imaging (MRI). The method used for isolation has been previously described [17]. NPCs were cultured in DMEM/F-12 medium (Hyclone) with 10% FBS (Gibco) and 1% penicillin/streptomycin. These cells were incubated at 37°C in a humidified atmosphere of 5% CO_2_.

### Characterization of iMSCs and sEVs

Surface antigens of iMSCs were detected using flow cytometry analysis. Cells were collected and incubated with 3% bovine serum albumin (Sigma-Aldrich) for 30 min to block any non-specific antigen binding. The cell suspensions were incubated with antibodies for iMSC-specific surface markers that included CD29, CD34, CD44, CD45, CD73, or HLA-DR (all from BD Biosciences) at 4°C for 30 min. The Guava easyCyte^TM^ flow cytometer (Millipore) was used to analyse the surface antigens.

iMSC-sEVs or FB-sEVs were isolated from the iMSC or FB culture supernatants, as previously described [18]. Briefly, after reaching 80% confluency, iMSCs and FBs were washed with PBS. The iMSCs were cultured with serum-free MSC medium (StemRD), and FBs were cultured in a high-glucose medium containing extracellular vesicle-depleted FBS (10%) for 48 h at 37°C in an atmosphere of 5% CO_2_. The medium was collected and centrifuged at 300×*g* for 10 min at 4°C to remove the remaining cells, followed by centrifugation at 2000×*g* for 10 min at 4°C to remove the dead cells, and another centrifugation at 10,000×*g* for 30 min at 4°C. The supernatant was ultra-centrifuged at 100,000×*g* for 70 min at 4°C. This step was repeated once. The sEVs were resuspended in PBS for use in the experiments. The morphology of sEVs was observed using transmission electron microscopy (TEM) by using a model H-7650 device (Hitachi) operating at an accelerating voltage of 80.0 kV. The concentration and size distribution of the sEVs were measured using nanoparticle analysis (NTA) by using ZetaView PMX 110 (Particle Metrix). The expression of characteristic markers of sEVs (CD9, CD63, Tsg101, GM130, and actin) were tested using western blotting.

### Establishment of the IVDD model and treatment in rats

Twelve-week-old Sprague–Dawley rats were used. All experimental procedures were approved by the Animal Research Committee of the First Affiliated Hospital of the University of Science and Technology of China. The rat model of IVDD was established as previously described [19]. Briefly, an experimental level rat tail disc (Co5/6) was punctured using a 20-gauge needle (IVDD group). The puncture was made through the centre of the disc to the opposite side, and the needle was rotated 180° and held for 10 s. Rats that were not treated were used as negative controls. One week after the initial surgery, 2 μL of sterile saline containing 1 × 10^10^ iMSC-sEVs/mL was injected into the punctured discs by using a 33-gauge needle, in the IVDD group. The negative group received an injection of FB-sEVs. The injections were repeated every 2 weeks. At week 8, MRI was performed on all rats, and the rats were euthanized for further analysis.

### MRI

After 8 weeks of the puncture procedure, all rats were examined using MRI examination to evaluate the degenerative changes in the sagittal T2-weighted images by using a 3.0 T clinical magnet (Siemens). T2-weighted sections in the median sagittal plane were obtained using the following settings: fast-spin echo sequence with a time to repetition of 5400 ms and a time to echo of 920 ms, 320 (h) 256 (v) matrix, field of view of 260, and four excitations. The section was 2 mm thick with a 0-mm gap. The degree of IVDD in the MR images was evaluated using the Pfirrmann grading system [20].

### Histological analysis

Rats were sacrificed and tail samples were fixed in 4% paraformaldehyde, decalcified in 10% EDTA, dehydrated using an alcohol gradient, and embedded in paraffin. The specimens were cut into 5-μm-thick sections. Haematoxylin–eosin (H&E) staining was performed to observe the IVDD. Briefly, all sections were deparaffinized, rehydrated, and stained in a haematoxylin solution for 5 min. After differentiation in 1% acid alcohol for 30 s, the sections were stained in an eosin solution for 30 s to 1 min. Sections were observed using optical microscopy. Immunofluorescence (IF) staining was used to detect the age-related P16 protein. The sections were deparaffinized, rehydrated, antigen retrieved, and blocked and incubated with primary antibody against P16 (1:200; Invitrogen) overnight at 4°C. The sections were incubated with Alexa Fluor 594-conjugated secondary antibody (1:400) for 1 h, and the nuclei were stained with 4′,6-diamidino-2-phenylindole (DAPI).

### Senescence-associated *β*-galactosidase (SA-*β*-gal) staining

SA-*β*-gal activity of NPCs was detected using a cellular senescence staining kit (Beyotime Biotechnology) according to the manufacturer’s instructions. Senescent cells were identified as blue-stained cells using phase-contrast microscopy. The proportion of positive cells was determined by counting the number of blue cells and dividing it by the number of cells observed.

### Proliferation assay

Cell proliferation was measured using a cell counting kit-8 (CCK-8, Dojindo Molecular Technologies). NPCs from different treatment groups were seeded onto 96-well plates at a density of 3000 cells per well. A volume of 10 μL of a CCK8-solution was added to 100 μL of medium and incubated for 2 h at 37°C. The absorbance was measured at 450 nm using a model 680 microplate reader (Bio-Rad).

### Western blotting analysis

Protein extracts were separated using sodium dodecyl sulphate-polyacrylamide gel electrophoresis (SDS-PAGE). The resolved proteins were transferred to polyvinylidene fluoride membranes. The membranes were blocked with 5% non-fat milk in Tris-buffered saline containing 0.1% Tween-20 for 2 h at room temperature. The membranes were then incubated with primary antibodies against the following proteins: P16 (1:1000; Abcam), collagen II (1:1000; Abcam), aggrecan (1:1000; Abcam), matrix metalloproteinase 3 (MMP3, 1:1000; Abcam), a disintegrin and metalloproteinase with thrombospondin motifs 4 (ADAMTS-4, 1:1000; Abcam), cAMP-specific 3′,5′-cyclic phosphodiesterase 4D (PDE4D, 1:1000; Abcam), Sirt6 (1:1000; Abcam), and actin (1:3000; Abcam) at 4°C overnight. Subsequently, the membranes were incubated with peroxidase-conjugated anti-rabbit IgG (1:3000; Abcam) or anti-mouse IgG (1:3000; Abcam) for 1 h at room temperature. Finally, the proteins were visualized using ECL (Thermo Fisher Scientific).

### qRT-PCR analysis

Extraction of sEV RNA was performed using Exoquick (QIAGEN). qRT-PCR was performed using the QuantiTect® SYBR Green PCR Master Mix. The default PCR settings used were 40 cycles at 94°C for 15 s, 55°C for 30 s, and 70°C for 30 s. Specific amplicons were identified using melting curve analysis.

### Uptake of iMSC-sEVs and FB-sEVs

The uptake of sEVs by NPCs was observed using a green fluorescent dye (DiO; Life Technologies) to label the iMSCs and FBs, according to the manufacturer’s instructions. The sEVs released by the labelled iMSCs or FBs were also labelled with DiO. The NPCs were cultured with a conditioned medium containing DiO-labelled sEVs, for 12 h.

### Transport of miRNA inhibitors into iMSC-sEVs

The miR-105-5p inhibitor and control miRNA inhibitor were purchased from QIAGEN. The miR-105-5p inhibitor and control miRNA inhibitor were transferred to iMSC-sEVs by using the Exo-Fect siRNA/miRNA Transfection Reagent Kit (SBI), according to the manufacturer’s instructions.

### Statistical analysis

The data are presented as means ± standard deviation. Statistical significance (*P* values) was determined using one-way analysis of variance or Student’s *t*-test. Statistical significance was set at *P* < 0.05.

## Results

### Characterization of iMSCs and sEVs derived from iMSCs or FBs

**Fig. 1:**
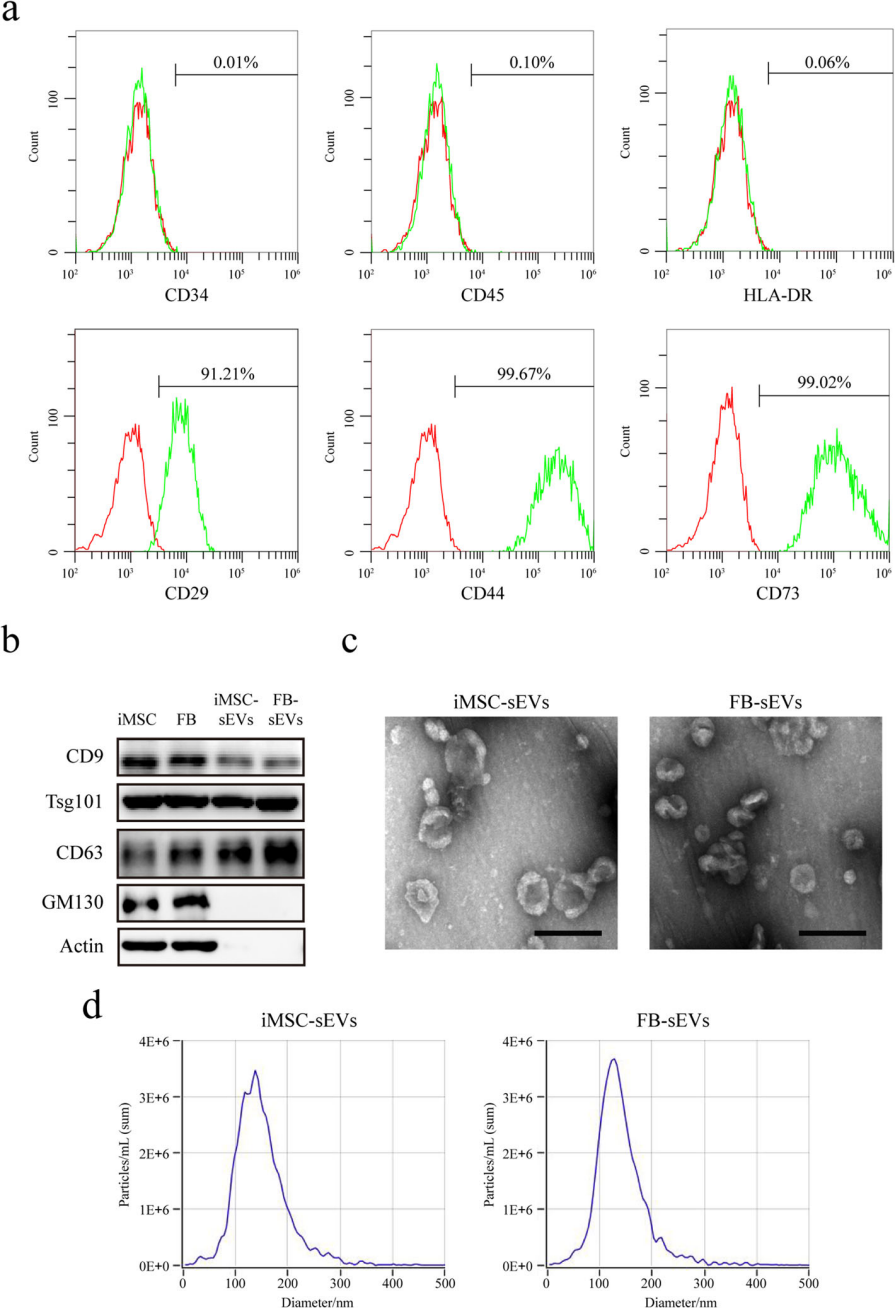
Characterization of iMSC and sEVs. **a** Flow cytometric analyses of phenotypic markers of iMSC. iMSCs expressed CD29, CD44, and CD73 and were negative for CD34, CD45, and HLA-DR. **b** Western blotting analysis indicated that sEVs expressed sEV markers, such as CD9, CD63, and TSG101, and were negative for GM130 and Actin. **c** Morphology of sEVs under TEM. Scale bar, 200 nm. **d** Particle size distribution of sEVs measured by NTA

iMSCs were successfully derived from iPSCs. As shown in previous studies [21], iMSCs expressed CD29, CD44, and CD73 and were negative for CD34, CD45, and HLA-DR (Fig. 1a). Next, we characterized the sEVs derived from iMSCs or FBs. Western blotting analysis indicated that the sEVs expressed sEV markers, such as CD9, CD63, and TSG101, and were negative for GM130 and actin (Fig. 1b). TEM revealed that the sEVs from iMSCs or FBs displayed a cup-shaped morphology (Fig. 1c). NTA showed that the sizes of the iMSC-sEVs and FB-sEVs ranged from 80 to 200 nm (Fig. 1d).

### Intradiscal injection of iMSC-sEVs ameliorates the progression of IVDD and the senescence of NPCs in a rat model

**Fig. 2:**
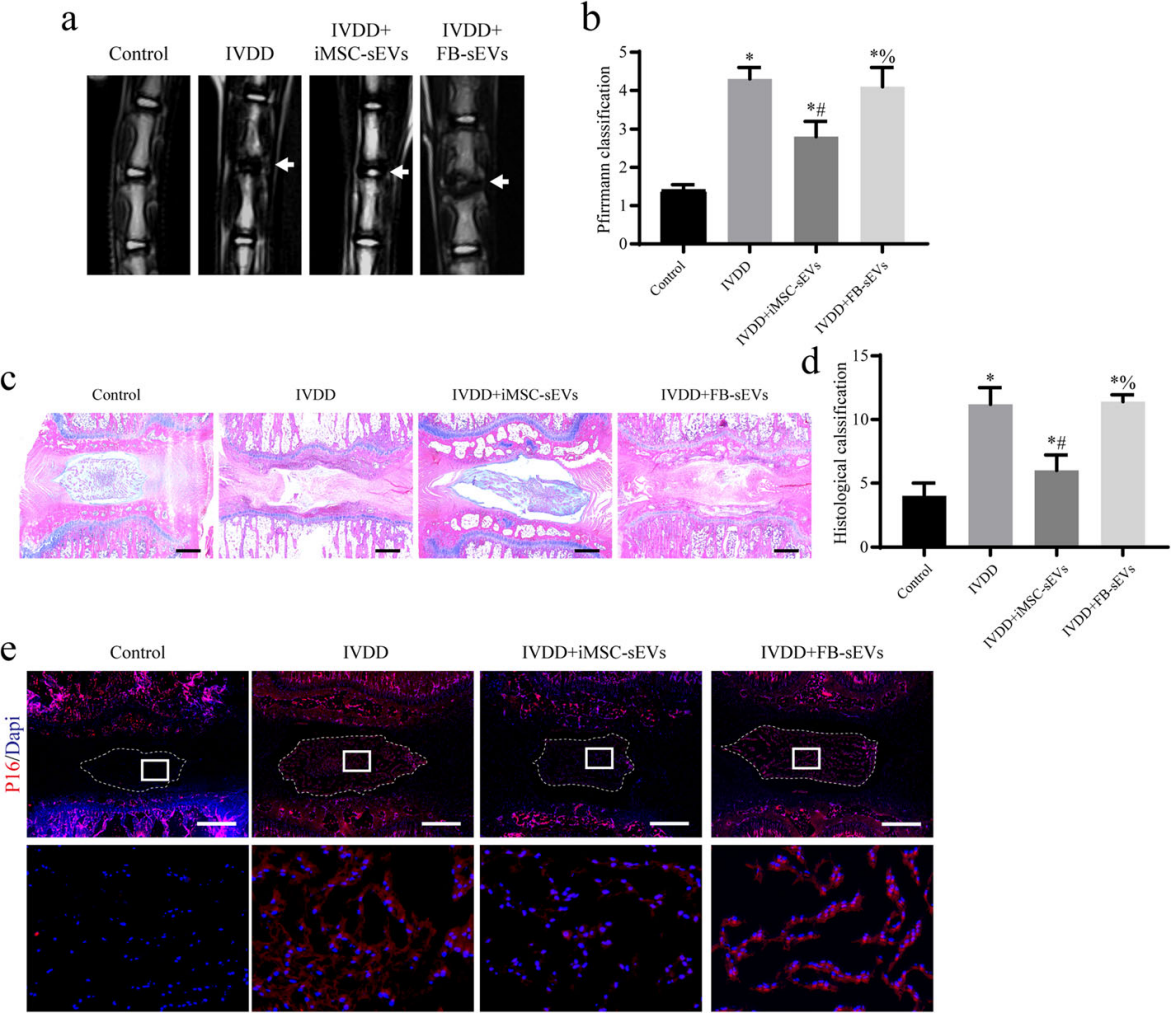
Intradiscal injection of iMSC-sEVs ameliorates the progression of IVDD and senescence of the NPCs in a rat model. **a** MRIs of the indicated groups were obtained at 8 weeks after needle puncture. **b** Pfirrmann MRI grade scores. **c** HE staining of IVDs in the indicated groups at 8 weeks after needle puncture. Scale bar, 1 mm. **d** The grading score of HE staining. **e** IF staining for P16 (red) at 4 weeks after needle puncture. DAPI was used to stain the nuclei. Scale bar, 1 mm. *P < 0.05 compared with the control group, ^#^*P* < 0.05 compared with IVDD group, ^%^*P* < 0.05 compared with IVDD+iMSC-sEVs group

A rat model of IVDD was successfully established 8 weeks after needle puncture. One week after the initial surgery, iMSC-sEVs were injected into the punctured disc for treatment. An FB-sEV injection was used as a negative control. MRI indicated that the T2-weighted signal intensities in the IVDD group were weaker than those in the control group. This change was ameliorated by iMSC-sEVs, while no significant improvement was observed using FB-sEVs (Fig. 2a). Pfirrmann MRI grading system, which indicates the degree of disc degeneration, demonstrated significantly higher scores in the IVDD group compared to those in the control group, and the scores decreased markedly after the application of iMSC-sEVs (Fig. 2b). Next, we observed the histological changes of the IVDs in each group using HE staining. The treatment with iMSC-sEVs remarkably reduced the loss of NPCs and restored the intervertebral height in a rat model of IVDD, while no significant differences were observed between the IVDD and FB-sEV injection groups (Fig. 2c, d). Previous studies found that the senescence of NPCs was closely related to the pathological progression of IVDD. Therefore, we further explored whether the therapeutic effects of iMSC-sEVs on IVDD involved the altered senescence of NPCs. IF staining for the age-related P16 protein indicated that iMSC-sEVs, but not FB-sEVs, significantly alleviated the senescence of NPCs 4 weeks after needle puncture (Fig. 2e). These results suggested that iMSC-sEVs significantly delayed the pathological progression of IVDD and ameliorated the senescence of NPCs in the rat model.

### iMSC-sEVs can ameliorate the senescent phenotype of NPCs and age-related dysfunction in vitro

**Fig. 3:**
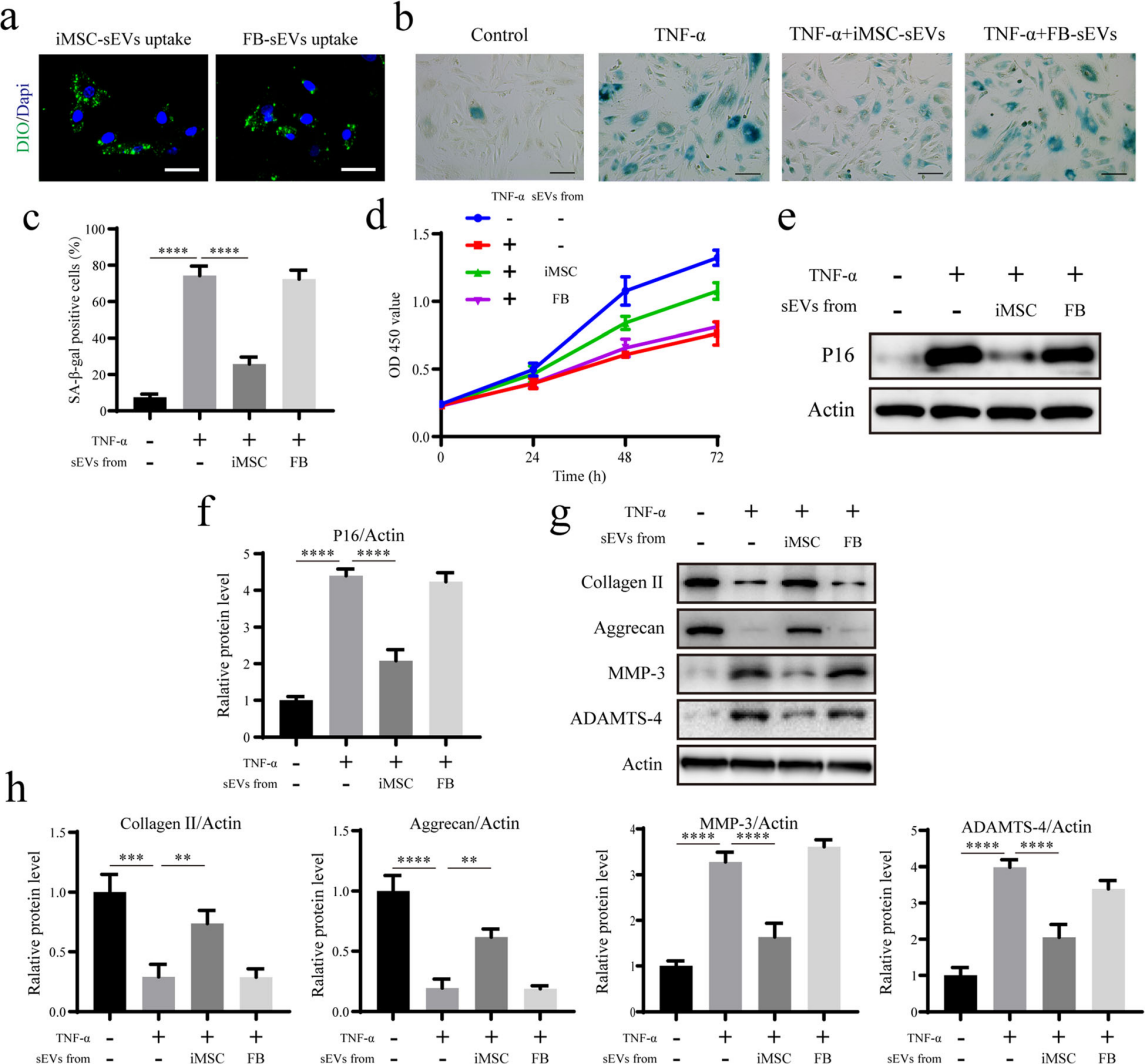
iMSC-sEVs can ameliorate the senescent phenotype of NPCs and age-related dysfunction in vitro. **a** IF analysis of DiO-labelled sEV internalization by NPCs. Scale bar, 50 um. **b** Representative micrographs of NPCs stained with SA-훽-gal in different treatment groups. SA-훽-gal-positive cells are shown in blue. Scale bar, 150 um. **c** Percentage of SA-훽-gal-positive cells. *n* = 3 per group. **d** CCK-8 analysis of NPC proliferation in different treatment groups. *n* = 5 per group. **e** The expression of P16 was assessed by Western blotting. **f** Densitometric quantification of the relative band intensity in **e**. *n* = 3 per group. **g** The expressions of collagen II, aggrecan, MMP-3, and ADAMTS-4 were assessed by Western blotting. **h** Densitometric quantification of the relative band intensity in **g**. *n* = 3 per group. *P<0.05, **P< 0.01, ***P< 0.001

To further investigate the effect of iMSC-sEVs on senescent NPCs, we first established an in vitro model of cellular senescence by treating NPCs with 10 ng/mL tumour necrosis factor-alpha (TNF-α) for 7 days [22]. The proportion of senescent NPCs and the expression level of age-related P16 protein increased, while the proliferation of NPCs decreased (Fig. 3b–f). Before examining the therapeutic effects of iMSC-sEVs on senescent NPCs, we first determined whether iMSC-sEVs or FB-sEVs could be endocytosed into senescent NPCs. DiO-labelled iMSC-sEVs or FB-sEVs were present in the perinuclear region after incubation for 12 h, suggesting an iMSC-sEV or FB-sEV uptake by senescent NPCs (Fig. 3a). To observe the effects of iMSC-sEVs on senescent NPCs, senescent NPCs were incubated with 1 × 10^10^ iMSC-sEVs/mL for 7 days. iMSC-sEVs significantly reduced the proportion of senescent NPCs (Fig. 3b, c). The CCK-8 assay demonstrated that iMSC-sEV treatment restored the proliferation of TNF-α-induced senescent NPCs (Fig. 3d). Western blot analysis also suggested that the expression of age-related P16 protein was significantly downregulated after iMSC-sEV treatment (Fig. 3e, f). Accordingly, the decreases in the levels of collagen II and aggrecan, which are anabolism markers of the extracellular matrix (ECM), and the increases in the levels of MMP-3 and ADAMTS-4 catabolism markers of the ECM were suppressed after senescent NPCs were treated with iMSC-sEVs, but not with FB-sEVs (Fig. 3g, h). These results revealed that iMSC-sEVs can ameliorate the senescent phenotype of NPCs and age-related dysfunction in vitro.

### iMSC-sEVs alleviate the senescence of NPCs by upregulating Sirt6 in vitro

**Fig. 4:**
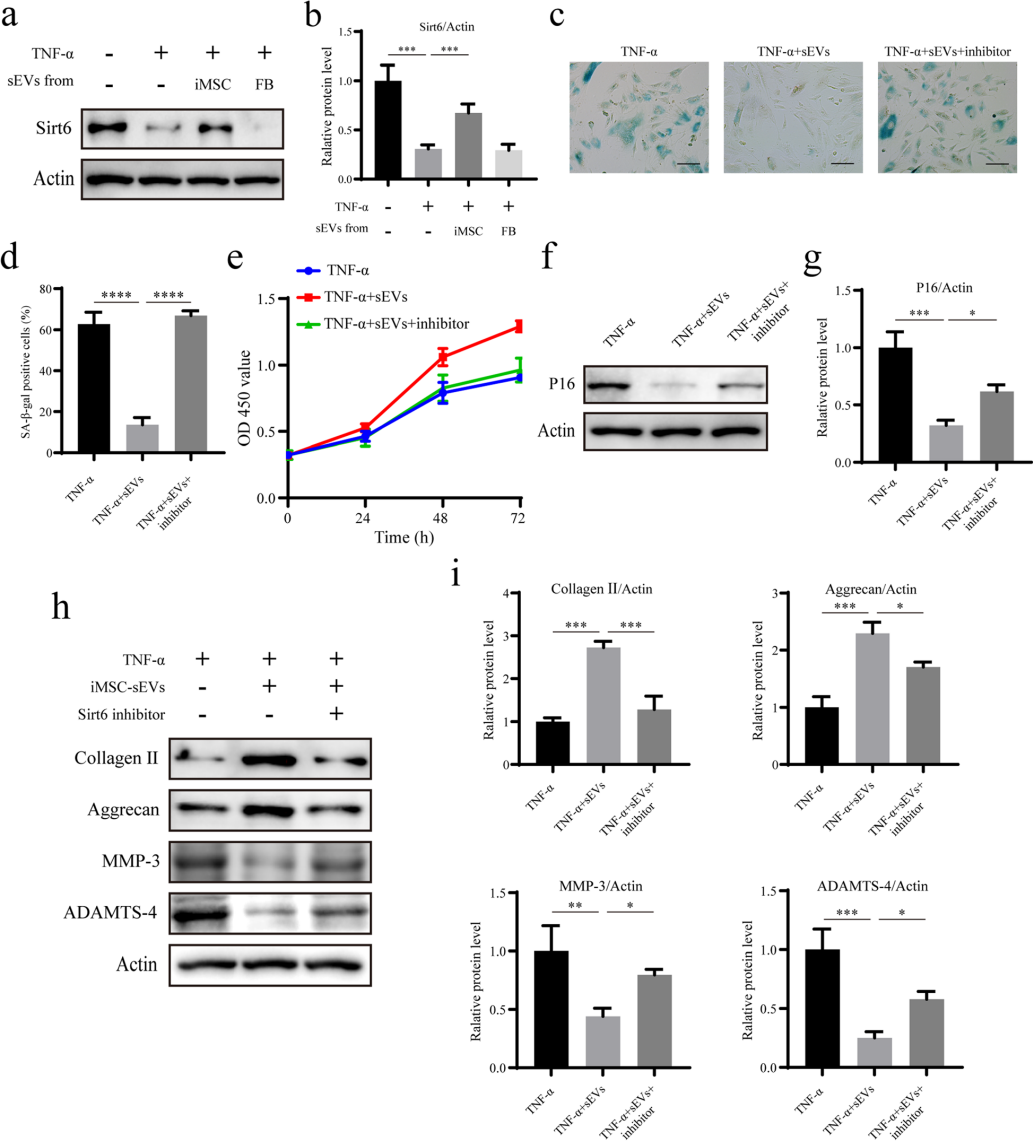
iMSC-sEVs alleviate senescence of NPCs by upregulating Sirt6 in vitro. **a** The expression of Sirt6 was assessed by Western blotting. **b** Densitometric quantification of the relative band intensity in **a**. *n* = 3 per group. **c** Representative micrographs of NPCs stained with SA-*β*-gal in different treatment groups. SA-*β*-gal-positive cells are shown in blue. Scale bar, 150 um. **d** Percentage of SA-*β*-gal-positive cells. *n* = 3 per group. **e** CCK-8 analysis of NPC proliferation in different treatment groups. *n* = 5 per group. **f** The expression of P16 was assessed by Western blotting. **g** Densitometric quantification of the relative band intensity in **f**. *n* = 3 per group. **h** The expressions of collagen II, aggrecan, MMP-3, and ADAMTS-4 were assessed by Western blotting. **i** Densitometric quantification of the relative band intensity in **h**. *n* = 3 per group. *P<0.05, **P< 0.01, ***P< 0.001

Sirt6 is a key protein that protects cells from senescence by more efficiently repairing the DNA double-strand breaks [23]. The expression level of Sirt6 can decrease with cellular senescence, and the reactivation of Sirt6 in senescent cells can alleviate cellular senescence and age-related dysfunction [24–26]. Therefore, we investigated whether Sirt6 is involved in the effects of iMSC-sEVs on senescent NPCs. Western blotting analysis showed that the expression of Sirt6 declined in senescent NPCs. iMSC-sEV treatment, but not FB-sEV treatment, recovered the expression of Sirt6 (Fig. 4a, b). To explore whether the upregulation of Sirt6 resulted in the therapeutic effects of iMSC-sEVs, we treated senescent NPCs with iMSC-sEVs and the Sirt6 inhibitor (OSS_128167). iMSC-sEVs failed to decrease the SA-훽-gal activity in senescent NPCs, when co-treated with the Sirt6 inhibitor (Fig. 4c, d), as well as the expression of P16 (Fig. 4f, g). The CCK-8 assay demonstrated that the Sirt6 inhibitor also abolished iMSC-sEV-mediated restoration of the proliferation ability in senescent NPCs (Fig. 4e). Accordingly, iMSC-sEVs mediated the upregulation of anabolism markers of the ECM (collagen II and aggrecan), and the downregulation of catabolism markers of the ECM (MMP-3 and ADAMTS-4) was obviously blocked by the Sirt6 inhibitor treatment (Fig. 4h, i). These results suggest that iMSC-sEVs can ameliorate the senescent phenotype of NPCs and age-related dysfunction in vitro through Sirt6 activation.

### iMSC-sEVs downregulate PDE4D expression and activate the Sirt6 signalling pathway by delivering miR-105-5p to senescent NPCs

**Fig. 5:**
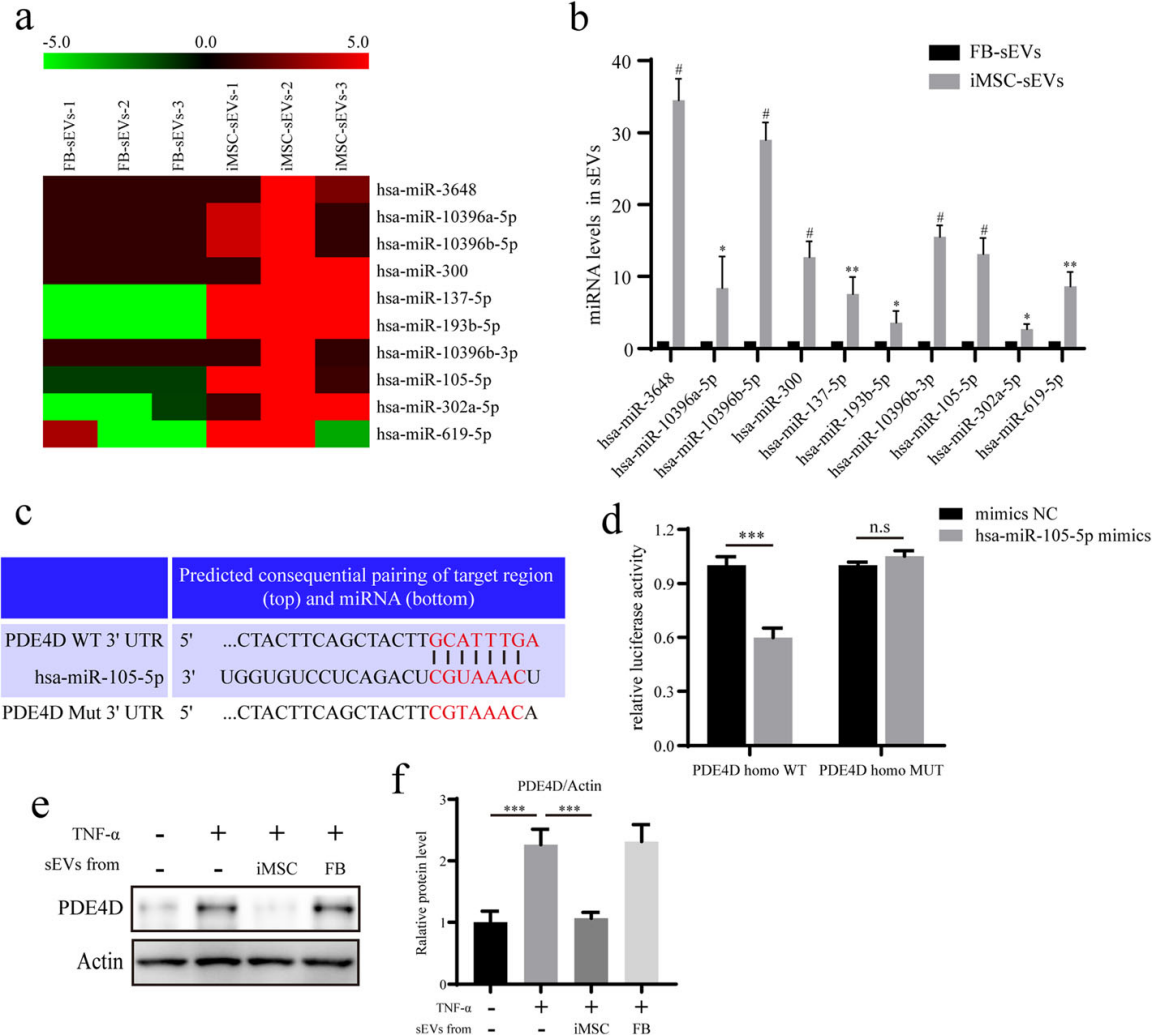
iMSC-sEVs downregulate PDE4D expression and activate the Sirt6 signalling pathway by delivering miR-105-5p into senescent NPCs. **a** Heatmap of the top 10 highest differential expressing miRNAs between iMSC-sEVs and FB-sEVs. **b** qRT-PCR validated the levels of the 10 upregulated miRNAs. **c** Schematic representation of the predicted PDE4D 3′-UTR indicating the has-miR-105-5p binding sites and the designed mutated versions of PDE4D 3′-UTR. **d** Luciferase activities in cells transfected with miR-105-5p mimics or negative control. **e** The expression of PDE4D was assessed by Western blotting. **f** Densitometric quantification of the relative band intensity in **e**. *n* = 3 per group. *P<0.05, **P< 0.01, ^#^*P*< 0.001; n.s, *P*> 0.05

Various miRNAs encapsulated in sEVs can be transported to the recipient cells and regulate gene expression post-transcriptionally by binding to the 3′-untranslated region (UTR) or the amino acid coding sequences of the target gene [27, 28]. To identify the miRNAs with therapeutic activity in iMSC-sEVs, we first performed a miRNA sequence analysis of the sEVs secreted by iMSCs or FBs. Ninety-two differentially expressed miRNAs were found in iMSCs-sEVs, compared with FB-sEVs. Given that no therapeutic effect on senescent NPCs was observed using FB-sEVs, we focused on the upregulated miRNAs in iMSCs-sEVs, compared with FB-sEVs. Ten miRNAs in iMSCs-sEVs were significantly upregulated compared with FB-sEVs (Fig. 5a). qRT-PCR results validated the levels of the 10 upregulated miRNAs (Fig. 5b). Next, we predicted the potential target genes of the 10 upregulated miRNAs by using the TargetScan and miRbase websites. Bioinformatic analysis demonstrated that miR-105-5p could bind to the 3′-UTR of PDE4D, which inactivates the Sirt6 pathway by decreasing the cellular levels of the second messenger, cAMP (Fig. 5c) [29]. A luciferase assay was used to validate whether miR-105-5p could directly target PDE4D. Luciferase activity was significantly decreased when transfected cells were incubated with miR-105-5p mimics. However, this effect was blocked by mutating the target sites in the 3′-UTR of PDE4D (Fig. 5d). Moreover, western blotting analysis also showed that the expression level of PDE4D in senescent NPCs treated with iMSCs-sEVs was significantly downregulated (Fig. 5e, f). These results suggested that iMSC-sEVs could deliver miR-105-5p to senescent NPCs and downregulate the expression level of PDE4D, subsequently activating the Sirt6 signalling pathway.

### Inhibition of miR-105-5p attenuates the therapeutic effect of iMSC-sEVs on senescent NPCs

**Fig. 6:**
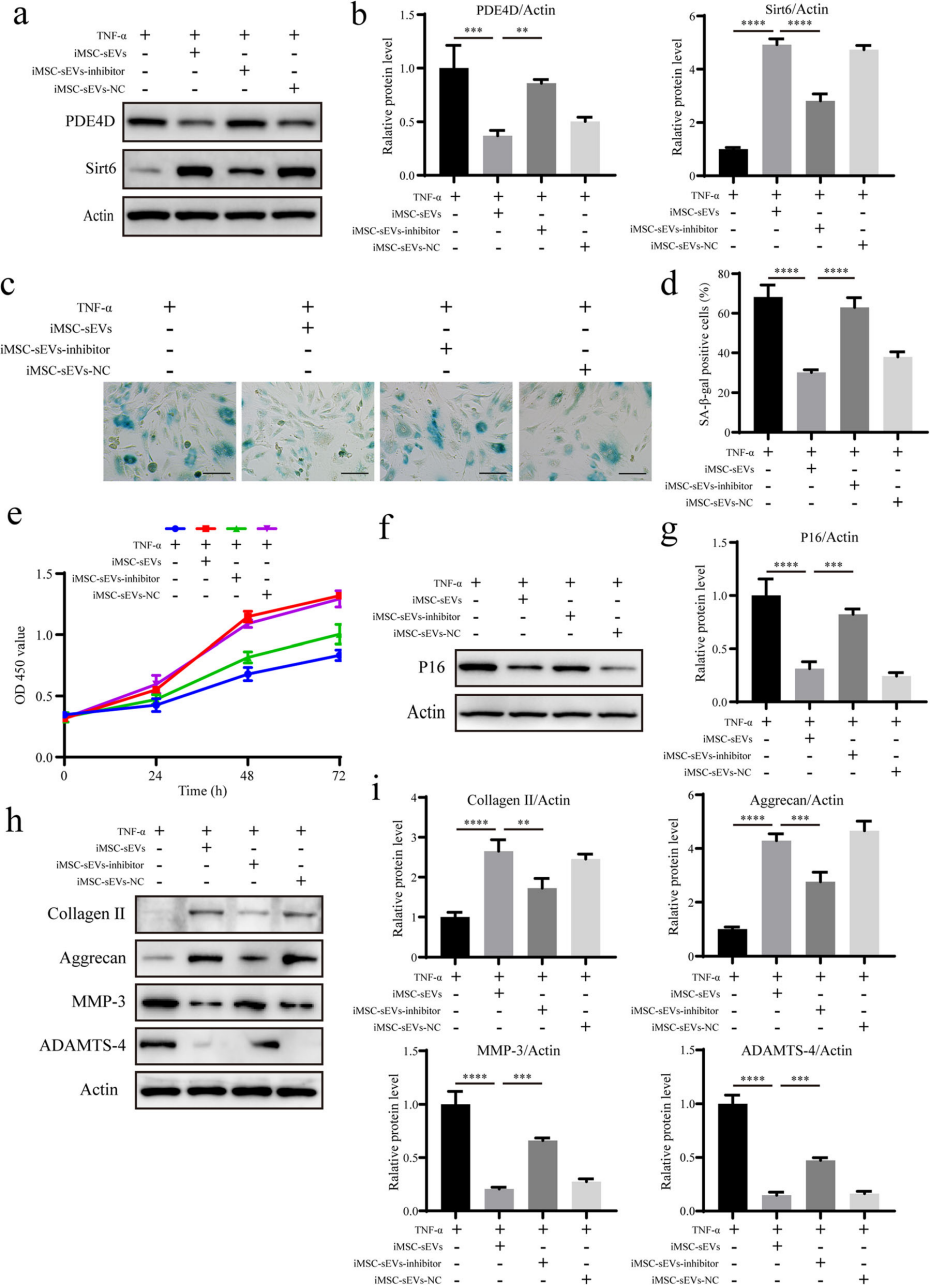
Inhibition of miR-105-5p attenuates the therapeutic effect of iMSC-sEVs on senescent NPCs. **a** The expressions of PDE4D and Sirt6 were assessed by Western blotting. **b** Densitometric quantification of the relative band intensity in **a**. *n* = 3 per group. **c** Representative micrographs of NPCs stained with SA-*β*-gal in different treatment groups. SA-*β*-gal-positive cells are shown in blue. Scale bar, 150 μm. **d** Percentage of SA-*β*-gal-positive cells. *n* = 3 per group. **e** CCK-8 analysis of NPC proliferation in different treatment groups. *n* = 5 per group. **f** The expression of P16 was assessed by Western blotting. **g** Densitometric quantification of the relative band intensity in **f**. *n* = 3 per group. **h** The expressions of collagen II, aggrecan, MMP-3, and ADAMTS-4 were assessed by Western blotting. **i** Densitometric quantification of the relative band intensity in **h**. *n* = 3 per group. *P<0.05, **P< 0.01, ***P<0.001

To further investigate whether miR-105-5p is crucial in the iMSC-sEV-mediated rejuvenation of senescent NPCs, the miR-105-5p inhibitor was incorporated into iMSC-sEVs. The binding of the inhibitor to miR-105-5p sequences abolished the biological function of miR-105-5p. We first examined the expression levels of PDE4D and Sirt6 after incubation of senescent NPCs with miRNA inhibitor-treated iMSC-sEVs. The expression of PDE4D was increased in the presence of miRNA inhibitor-treated iMSC-sEVs, whereas the expression of Sirt6 was decreased (Fig. 6a, b). These findings indicated that the inhibition of miR-105-5p promoted the activation of the Sirt6 signalling pathway. SA-*β*-gal staining and the CCK-8 assay revealed that the inhibition of miR-105-5p significantly abolished the iMSC-sEV-mediated reduction of the proportion of senescent NPCs and the restoration of the proliferation capacity (Fig. 6c–e). The level of age-related P16 protein also increased when senescent NPCs were treated with miRNA inhibitor-treated iMSC-sEVs (Fig. 6f, g). Additionally, the levels of collagen II and aggrecan, anabolism markers of the ECM, decreased, and the levels of the MMP-3 and ADAMTS-4 catabolism markers of the ECM increased upon treatment with miRNA inhibitor-treated iMSC-sEVs (Fig. 6h, i). These results demonstrated that the inhibition of miR-105-5p attenuated the therapeutic effect of iMSC-sEVs on senescent NPCs in vitro.

### PDE4D overexpression in NPCs blocks the therapeutic effect of iMSC-sEVs on senescent NPCs

**Fig. 7:**
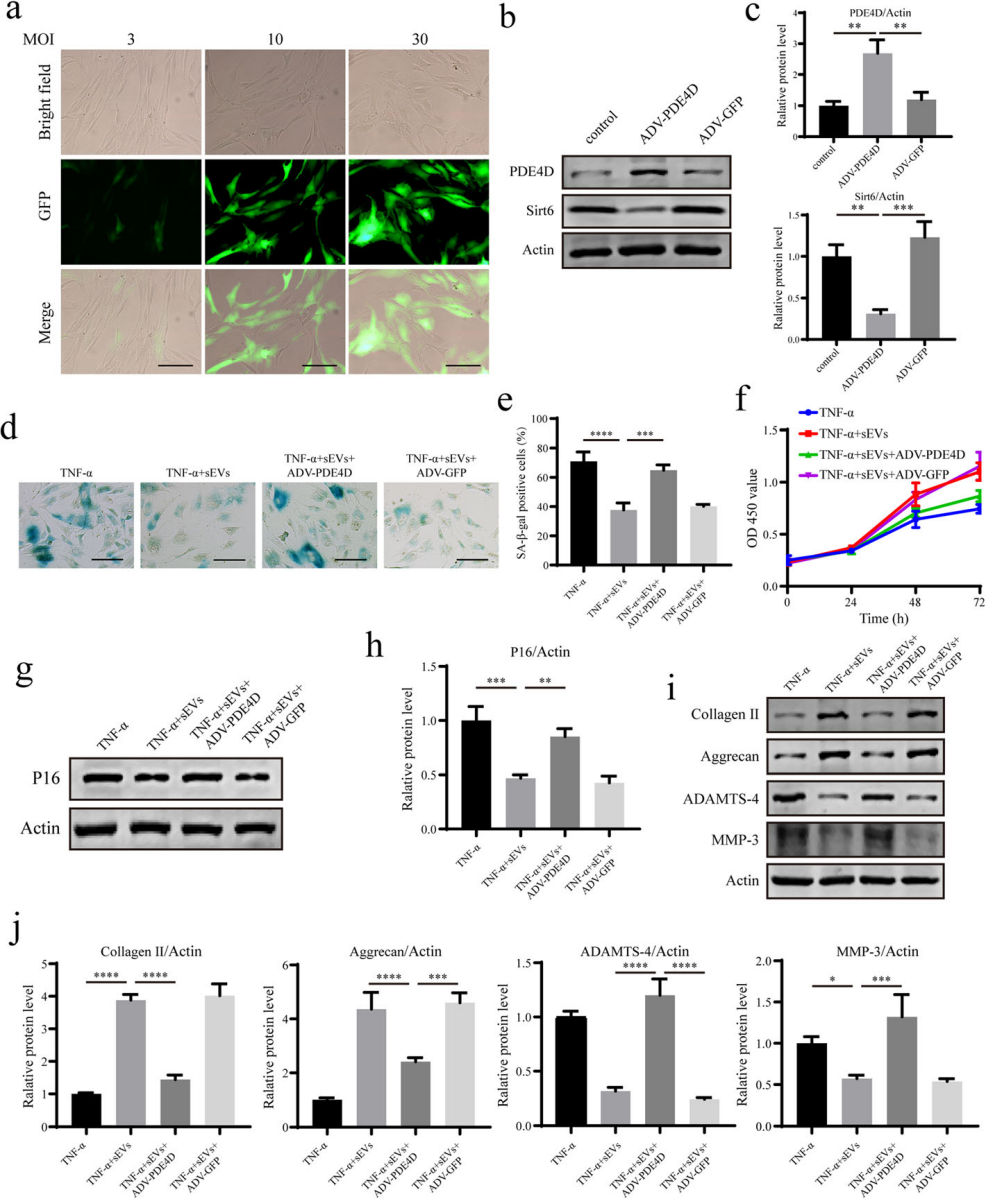
PDE4D overexpression in NPCs blocks the therapeutic effect of iMSC-sEVs on senescent NPCs. **a** IF analysis of NPCs transfected with GFP-labelled adenovirus under different MOI values. Scale bar, 150 um. **b** The expressions of PDE4D and Sirt6 were assessed by Western blotting. **c** Densitometric quantification of the relative band intensity in **b**. *n* = 3 per group. **d** Representative micrographs of NPCs stained with SA-*β*-gal in different treatment groups. SA-*β*-gal-positive cells are shown in blue. Scale bar, 150 um. **e** Percentage of SA-*β*-gal-positive cells. *n* = 3 per group. **f** CCK-8 analysis of NPC proliferation in different treatment groups. *n* = 5 per group. **g** The expression of P16 was assessed by Western blotting. **h** Densitometric quantification of the relative band intensity in **g**. *n* = 3 per group. **i** The expressions of collagen II, aggrecan, MMP-3, and ADAMTS-4 were assessed by Western blotting. **j** Densitometric quantification of the relative band intensity in **i**. *n* = 3 per group. *P<0.05, **P< 0.01, ***P<0.001

To examine the key role of PDE4D in the therapeutic effect of miR-105-5p incorporated in iMSC-sEVs, a PDE4D overexpressing adenovirus was synthesized and transfected into NPCs. We first explored the optimal multiplicity of infection (MOI) value for the adenovirus transfection of NPCs. When the MOI was 10, the efficiency of the adenovirus transfection into NPCs was almost 100%, and the efficiency of the adenovirus transfection was not significantly improved when the MOI continued to increase (Fig. 7a). Therefore, a MOI of 10 was used in the following experiments. Western blot analysis showed that the expression level of PDE4D protein could be significantly upregulated when the adenovirus was transfected into NPCs; however, Sirt6 activation in NPCs with a PDE4D overexpressing adenovirus was decreased (Fig. 7b, c). Next, we treated NPCs with TNF-α and PDE4D-overexpressing adenovirus and observed an age-related phenotype 7 days later. iMSC-sEV-mediated reduction in the proportion of senescent NPCs, the restoration of the proliferation capacity, and the downregulation of the level of age-related P16 protein were significantly attenuated (Fig. 7d–h). Accordingly, iMSC-sEV-mediated increases in the levels of the anabolism markers of the ECM and the decreases in the catabolism markers of the ECM were suppressed after overexpressing PDE4D (Fig. 7i, j). The results indicated the therapeutic activity of miR-105-5p transmitted by iMSC-sEVs by targeting the downregulation of PDE4D expression.

## Discussion

During disc degeneration, disorders in the physiological behaviour of NPC cells and ECM synthesis eventually lead to the biomechanical impairment of the IVD. Previous studies have suggested that the senescence of NPCs is involved in these pathological changes [30], indicating that targeting senescent NPCs would be an effective treatment strategy for IVDD [31, 32]. Recent evidence suggests that MSC transplantation into degenerated discs may be beneficial due to the paracrine function of MSCs. Among these paracrine bioactive substances, sEVs are attracting increasing interest. sEVs are a class of natural nanoparticles enclosed by a lipid bilayer with a diameter of 30–150 nm. sEVs can deliver their internal contents of parental cells, including proteins, nucleic acids, and lipids, into the target cells, resulting in metabolic changes in the recipient cells. sEVs pose no tumorigenesis or immune response risks. MSC-derived sEVs may be capable of alleviating cellular senescence. However, challenges remain in the large-scale preparation of sEVs derived from MSCs. The quantity and quality of MSC-derived sEVs are hindered by drawbacks that include the limited proliferative capacity of MSCs in vitro, MSC donor age, and the invasive MSC harvesting procedures required. One solution to these problems may be the use of MSCs derived from iPSCs. Numerous studies have found that iMSCs resemble some conventional MSCs derived from bone marrow or adipose tissue in terms of both phenotype and function [13, 33]. The potential advantages of iMSCs have been described previously [13].

Recent studies have shown the promising therapeutic effects of iMSC-sEVs in tissue repair, including skin wound healing, osteoarthritis, bone defects, and limb ischemia [21, 34–36]. Consistent with these results, here, we demonstrated that intervertebral injections of iMSC-sEVs can significantly delay IVDD in rats, as well as the senescence of NPCs. In addition, in vitro experiments also showed that iMSC-sEVs remarkably alleviated the senescent phenotype of NPCs and age-related dysfunction.

DNA damage has long been implicated in cellular senescence. Unresolved DNA damage can accelerate cellular senescence and promote disease development [37]. DNA repair is regulated by DNA damage response genes. *Sirt6* is one gene that responds to DNA repair and promotes longevity [38]. The expression level of *Sirt6* decreases with cellular senescence and the reactivation of Sirt6 in senescent cells can alleviate cellular senescence and age-related dysfunction due to the more efficient DNA double-strand break repair [23, 24]. Here, to investigate whether Sirt6-mediated signalling is involved in the effects of iMSCs-sEVs on NPC senescence, the level of Sirt6 was detected. The effects of iMSC-sEVs on senescent NPCs were evidenced by the activation of the Sirt6 signalling pathway.

Accumulating evidence indicates that sEVs are rich in a variety of miRNAs. The miRNAs encapsulated in sEVs can be transferred to recipient cells and can regulate their function by regulating gene expression post-transcriptionally [39]. However, few studies have examined miRNAs in iMSCs-sEVs. Presently, we provide the first piece of evidence of the differential expression of miRNAs in iMSCs-sEVs using miRNA sequence analysis. There was no evidence of an effect of FB-sEVs on senescent NPCs. Thus, it seems reasonable to speculate that the crucial miRNAs that show therapeutic effect should be upregulated in iMSCs-sEVs. Among these upregulated miRNAs, miR-105-5p was predicted to bind the 3′-UTR of PDE4D, as observed using TargetScan and miRDB gene prediction websites, which scrutinize high scoring target genes. PDE4D is a cAMP-specific hydrolase. The inhibition of PDE4D activity could increase the cAMP concentration, which leads to AMP-activated phosphate kinase (AMPK) and Sirt6 activation [40], the eventual alleviation of age-related phenotypes, and an extended lifespan. PDE4 inhibitors and cAMP analogues may protect against ageing-related diseases, such as Alzheimer’s disease [41]. To confirm the crucial role of miR-105-5p on the PDE4D-Sirt6 axis, we inhibited the biological function of miR-105-5p in iMSCs-sEVs. This inhibition abolished the therapeutic effect of iMSC-sEVs on senescent NPCs, increased the expression levels of PDE4D, and reduced the expression levels of Sirt6. In addition, the overexpression of PDE4D in senescent NPCs also blocked the therapeutic effect of iMSC-sEVs on senescent NPCs. These data reveal that iMSC-sEV-mediated miR-105-5p transfer leads to higher cAMP concentrations by targeting PDE4D and activating Sirt6 pathway, a key cascade signalling pathway involved in IVDD.

## Conclusions

This is the first study to evaluate the therapeutic effects of iMSC-sEVs on IVDD. iMSC-sEVs could rejuvenate the senescence of NPCs and attenuate the development of IVDD. iMSC-sEVs exerted their anti-ageing effects by delivering miR-105-5p to senescent NPCs and activating the Sirt6 pathway, which is a pivotal pathway in the response to DNA repair and promotes longevity. Our findings also indicate that iMSCs are a promising MSC candidate for obtaining sEVs at a large scale, while avoiding several defects related to the present applications of MSCs, and that iMSC-sEVs could be a novel cell-free therapeutic tool for the treatment of IVDD. Notably, the effects of iMSC-sEVs on Sirt6 activation and age-related dysfunction were not entirely blocked by miR-105-5p antagomir treatment (Fig. 6c–i). This suggests that other molecular mechanisms might also be involved and requires further exploration.

## Data Availability

The datasets used and/or analysed during the current study are available from the corresponding author upon reasonable request.

## Abbreviations

IVDD: Intervertebral disc degeneration
NPCs: Nucleus pulposus cells
MSC: Mesenchymal stem cell
sEVs: Small extracellular vesicles
iPSCs: Induced pluripotent stem cells
iMSCs: MSCs derived from induced pluripotent stem cells
iMSC-sEVs: iMSC-derived small extracellular vesicles
DMEM: Dulbecco’s modified Eagle medium
FBS: Foetal bovine serum
FBs: Fibroblasts
MRI: Magnetic resonance imaging
TEM: Transmission electron microscopy
NTA: Nanoparticle analysis
H&E: Haematoxylin–eosin
IF: Immunofluorescence
SA-*β*-gal: Senescence-associated *β*-galactosidase
CCK-8: Cell counting kit-8
MOI: Multiplicity of infection
